# Preoperative systemic immune-inflammation index as a prognostic indicator for patients with urothelial carcinoma

**DOI:** 10.3389/fimmu.2023.1275033

**Published:** 2023-11-20

**Authors:** Jianxiong Zheng, Lei Peng, Shaohua Zhang, Haiyang Liao, Jiayao Hao, Song Wu, Haili Shen

**Affiliations:** ^1^ The Second Clinical Medical College, Lanzhou University, Lanzhou, China; ^2^ Department of Rheumatology and Immunology, Affiliated Hospital of North Sichuan Medical College, Nanchong, China; ^3^ South China Hospital, Health Science Center, Shenzhen University, Shenzhen, China; ^4^ Department of Rheumatology and Immunology, Second Hospital of Lanzhou University, Lanzhou, China

**Keywords:** systemic immune-inflammation index, SII, urothelial carcinoma, bladder carcinoma, upper tract urothelial carcinoma, meta-analysis, prognostic factors

## Abstract

**Background:**

The systemic immune-inflammation index (SII) is a cost-efficient indicator for carcinoma prognosis. However, its utility in urothelial carcinoma (UC) prognosis is disputed. This meta-analysis aims to assess SII’s prognostic value in UC.

**Methods:**

A thorough search of databases including PubMed, Web of Science, Embase, Cochrane Library, and Scopus, was conducted to find studies until January 11, 2023. Eligibility criteria were applied to select studies. Hazard ratios (HRs) and 95% confidence intervals (CIs) were extracted from selected studies and compiled in a meta-analysis to gauge SII’s association with survival outcomes such as overall survival (OS), cancer-specific survival (CSS), recurrence-free survival (RFS), and progression-free survival (PFS).

**Results:**

This analysis includes 19 studies with 12505 UC patients. It was found that high SII significantly correlated with worse OS in UC patients (HR 1.430, 95% CI 1.237-1.653, P<0.001). High SII values also linked with poorer CSS (HR 1.913, 95% CI 1.473-2.485, P<0.001), RFS (HR 1.240, 95% CI 1.097-1.403, P=0.001), and PFS (HR 1.844, 95% CI 1.488-2.284, P<0.001) compared to low SII values. Subgroup analysis revealed SII’s consistent prognostic value in UC across races, carcinoma types, sample sizes, and SII cut-off values, suggesting its potential as a prognostic indicator in UC patients.

**Conclusion:**

Current evidence suggests SII as a promising, cost-efficient predictor in UC patients. This meta-analysis indicates SII’s potential as a valuable prognostic tool in UC patients.

**Systematic review registration:**

https://www.crd.york.ac.uk/PROSPERO/display_record.php?RecordID=307643, identifier CRD42022307643.

## Introduction

Uroepithelial carcinoma (UC) is the most prevalent urological carcinoma, encompassing upper urinary tract urothelial carcinoma (UTUC) and bladder carcinoma (BC) (1). According to global carcinoma statistics, BC alone accounted for an additional 573,278 new cases and 212,536 new deaths in 2020 (2). UTUC, which represents around 5-10% of all UC cases, has an estimated incidence rate of 1-2 cases per 100,000 person-years in Western countries (3, 4). Although UTUC and BC have distinct features, they share similar morphological structure and carcinoma biology, and have been recognized as a homogenous disease entity until recently (5). These carcinomas tend to recur after initial treatment, making UC a challenging carcinoma to manage (6). Presently, there is a dearth of clear biochemical markers that can predict the clinicopathological features and prognosis of UC. Consequently, there is a pressing need to identify a simple and cost-effective indicator that can not only detect the clinical characteristics of carcinoma prior to surgery but also aid in predicting the prognosis of UC patients. Such an indicator would greatly assist clinicians and patients in making informed decisions regarding treatment plans.

The association between inflammation and cancer is profoundly intertwined, primarily manifested through two distinct pathways (7, 8). The first pathway is instigated by genetic factors, encompassing gene mutations and chromosomal rearrangements. These genetic modifications trigger the activation of specific oncogenes and the suppression of cancer-inhibiting genes. As cells undergo transformation, they commence the secretion of inflammatory mediators, instigating the development of an inflammatory microenvironment in tissues initially devoid of inflammation. The second pathway is propelled by inflammatory conditions that augment the susceptibility to cancer. Within this extrinsic pathway, inflammation or infection can amplify the risk of cancer in specific anatomical locales. The convergence of these two pathways culminates in the activation of transcription factors within neoplastic cells, principally featuring nuclear factor κB (NF-κB), signal transducer and activator of transcription 3 (STAT3), and hypoxia-inducible factor 1α (HIF1α). This activation consequently engenders the production of inflammatory cytokines, including IL-8, IL-10, and TNFα.

Understanding the relationship between preoperative inflammation-based scores and carcinoma prognosis would aid in more efficient follow-up surveillance. By identifying and assessing inflammation markers before surgery, healthcare professionals can better predict carcinoma progression and outcomes, enabling targeted surveillance strategies and personalized treatment plans to improve patient outcomes (8, 9). Several inflammation-based scores, such as neutrophil-to-lymphocyte ratio (NLR), platelet-lymphocyte ratio (PLR), lymphocyte-monocyte ratio (LMR), and lymphocyte-C-reactive protein ratio (LCR), have been studied as potential prognostic factors for carcinoma. However, their predictive value for UC still has several limitations and deficiencies (10). Recently, the Systemic Immune-inflammation Index (SII) has gained attention as a novel inflammatory marker. It is calculated by multiplying the platelet count with the neutrophil count and dividing it by the lymphocyte count. Compared to other inflammatory markers, SII is considered to have superior prognostic value in assessing inflammation-related conditions (11, 12). The SII incorporates three peripheral blood inflammatory biomarkers, providing a more comprehensive reflection of the balance between inflammation and immune response in the body. While some studies have suggested a correlation between SII and poor prognosis in UC, the evidence remains controversial and uncertain (13–15). Hence, additional evidence-based research is warranted to comprehensively evaluate the prognostic value of SII in UC. This would provide robust evidence to support prognostic assessments in UC and assist clinicians and patients in making more informed treatment decisions.

## Materials and methods

### Protocol

In this study, we followed the 2020 Preferred Reporting Items for Systematic Reviews and Meta-Analyses (PRISMA) guideline (16) and were registered in PROSPERO (CRD42022307643, website link: https://www.crd.york.ac.uk/PROSPERO/display_record.php?RecordID=307643).

### Literature search

We conducted a comprehensive search of electronic databases, including PubMed, Web of Science, Embase, Cochrane Library, and Scopus, to identify studies published in the English language from inception to April 12, 2023. The search strategy utilized the following keywords: (systemic immune-inflammation index OR SII) AND (bladder carcinomas OR bladder cancer OR upper tract urothelial cancer OR upper tract urothelial carcinoma OR Urothelial tumor OR Urothelial carcinoma OR Urothelial cancer OR ureter cancer OR urethral carcinoma OR ureteral carcinoma OR carcinoma of renal pelvis OR Urothelial carcinoma OR carcinoma of the urothelium) AND (prognosis OR outcome OR mortality OR survival OR recurrence OR metastasis OR progression). In addition, we reviewed the references of relevant reviews and meta-analyses to supplement the identified citations. Any discrepancies encountered during the search results were resolved through discussion.

### Selection criteria

Studies were included based on the following criteria: (1) patients who had undergone histopathological diagnosis of urothelial carcinoma; (2) the studies presented hazard ratios (HRs) and corresponding 95% confidence intervals (CIs) elucidating the association between preoperative SII and survival outcomes, including Overall Survival (OS), cancer-specific survival (CSS), recurrence-free survival (RFS), and/or Progression-free survival (PFS); (3) the studies provided a defined threshold value for preoperative SII. Exclusion criteria encompassed: (1) fundamental research or studies involving animal models; (2) reviews, meta-analyses, comments, meeting reports, case reports, letters, and unpublished research; (3) studies lacking sufficient or inaccessible data; and (4) duplicate publications.

### Data extraction and quality assessment

Independently, Lei Peng and Jianxiong Zheng assessed each included study with the Cochrane Newcastle–Ottawa Scale (9 points highest score) (17). Scores from 7 to 9 are considered to be of high quality in this meta-analysis. In addition, all of the survival outcomes were directly presented as HRs and corresponding 95% CIs. The primary outcome of this meta-analysis was the OS, and the secondary outcomes were CSS, RFS, and PFS. Data from multivariate analysis were used when the data in a study had been analyzed in two ways simultaneously.

### Statistical analyses

Pooled HRs with corresponding 95% CIs to evaluate the relationship between preoperative SII and survival outcomes in this meta-analysis. Cochran’s Q and Higgin’s I^2^ tests were employed to assess the heterogeneity. A random-effects model was used for pooling analysis. Additionally, subgroup analysis was conducted to investigate potential sources of heterogeneity and sensitivity analyses were also conducted to assess the effect of individual study data on survival outcomes. Publication bias was assessed using Begg’s test and funnel plot. All statistical analyses were performed using Stata version 17 and *P*-value less than 0.05 was considered statistically significant.

## Results

### Study characteristics

**Figure 1:**
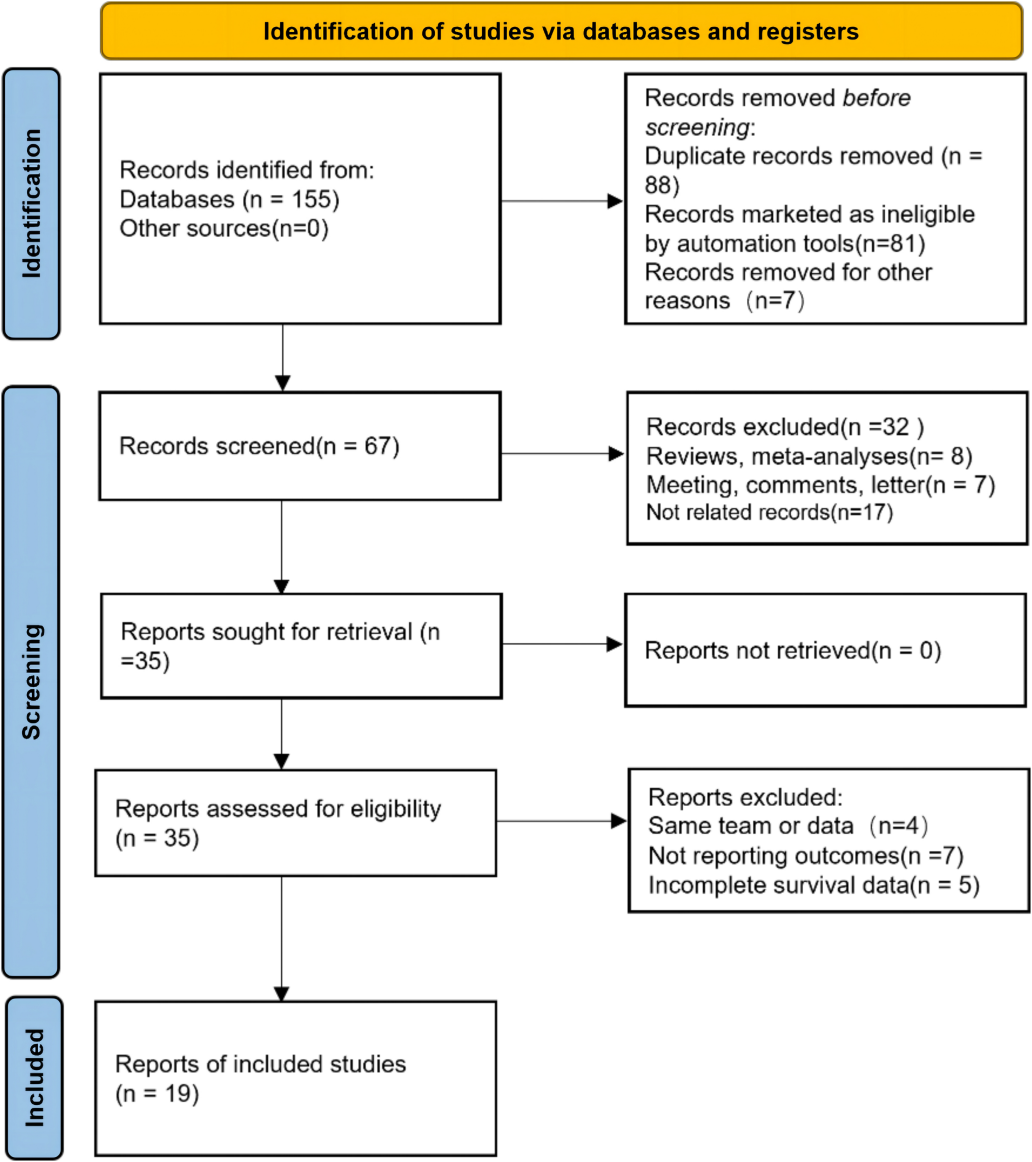
Flow diagram of studies selection process.

Our study selection process is illustrated in **Figure 1**. The primary data search retrieved a total of 155 articles based on the search strategy, and 67 studies remained after duplicate publications were removed. After having read the titles and abstracts, 35 potentially eligible papers underwent full-text reviewing. Finally, 19 studies that comprised 12505 patients were included in this meta-analysis (11, 13–15, 18–32). All the included studies have been published within the last 5 years (2019–2023); 13 studies focused on BC, 5 studies about UTUC, and the last one was on mixed (BC and UTUC). Notably, all of the 19 studies were retrospective, and 12 of them had been conducted in China, 3 in multi-country, 2 in Japan, and 2 in Italy and Turkey. The sample size in the included studies ranged from 70 to 4,335 patients, with the median patient age ranging from 59 to 73 years, and the cutoff values of SII ranged from 276.685 to 1375. 13 studies (15 datasets) reported the association between SII and OS, 9 studies (10 datasets) between SII and CSS, and 11 studies (12 datasets) investigated associations between SII and RFS, and 4 studies reported the association between SII and PFS. None of the studies had a NOS score below 7, indicating that the overall quality of the included studies was high. The main features of the included studies are presented in **Table 1**.

**Table 1:** Baseline characteristics of include studies and methodological assessment.

	Author	Year	Country	Study design	Sample size	Cancer type	Intervention	Age, year	Cut-off	Follow-up time, Months	Analysis method	Survival analysis	NOS Quality score	References
1	Mori, et al.	2021	Multicountry	Retrospective	2373	UTUC	RNU	69 ^a^	485	38^a^	Multivariate	OS、CSS、RFS	8	(11)
2	Chien, et al.	2021	China	Retrospective	376	UTUC	RNU	69.0 (15)^b^	485	52.0 (16.7) ^b^	Multivariate	CSS	8	(21)
3	Zheng, et al. (VC)	2020	China	Retrospective	272	UTUC	RNU	65.87 ± 10.35^e^	672.44	44.6 (26.8-65.3) ^b^	Multivariate	OS、CSS、RFS	7	(27)
4	Zheng, et al. (TC)	2020	China	Retrospective	253	UTUC	RNU	67.59 ± 10.49^e^	672.44	33.8 (16.7-64.4) ^b^	Multivariate	OS、CSS、RFS	7	(27)
5	Katayama,et al.	2021	Multicountry	Retrospective	1117	BC	TURBT	M 67 (I 58–74) ^b^	580	64 (26–110) ^b^	Multivariate	OS、CSS、PFS、RFS	8	(24)
6	Jan, et al.	2019	China	Retrospective	424	UTUC	RNU	M 70 (I 62–77) ^b^	580	35 (14–60) ^b^	Multivariate	OS、CSS、PFS	7	(23)
7	Kobayashi, et al.	2021	Japan	Retrospective	103	UTUC	RNU	M 73 (I 68–78) ^b^	520	41 (20–60) ^b^	Multivariate	OS、CSS	8	(15)
8	Yamashita, et al.	2021	Japan	Retrospective	237	BC	RC	M 73 (I 67–79) ^b^	438	38(17–64) ^b^	Multivariate	OS、CSS	7	(28)
9	Tang, et al.	2020	China	Retrospective	79	BC	RC	63:62 ± 7:52 ^e^	547.3	31 ^a^	Univariate	OS、RFS	7	(13)
10	Zhao, et al.	2021	China	Retrospective	216	BC	TURBT	59 (25– 87) ^d^	276.685	59.41 (2– 89) ^c^	Multivariate	RFS	7	(26)
11	Fornarini, et al.	2021	Italy	Retrospective	267	UC	Immunotherapy	M 69 (I 62-74) ^b^	1375	9.5 (95% CI 8.8-10.4) ^b^	Multivariate	OS、PFS	7	(22)
12	Bi, et al.	2020	China	Retrospective	387	BC	TURBT and BCG	71 (34–89) ^d^	467.76	108 (5–191) ^d^	Multivariate	OS、CSS	7	(20)
13	Yılmaz, et al.	2020	Turkey	Retrospective	152	BC	RC	66 (43–88) ^b^	768	16 (1–209) ^d^	Multivariate	OS、PFS	8	(25)
14	Grossmann, et al.	2021	Multicountry	Retrospective	4335	BC	RC	67 (60, 73) ^b^	610	42 (18–85) ^b^	Multivariate	OS、CSS、RFS	7	(29)
15	Zhang, et al. (PV)	2019	China	Retrospective	139	BC	RC	67 (29–87) ^d^	507	NR	Multivariate	OS	7	(14)
16	Zhang, et al. (VC)	2019	China	Retrospective	70	BC	RC	66 (37–87) ^d^	507	NR	Multivariate	OS	7	(14)
17	Ke, et al.	2021	China	Retrospective	184	BC	TURBT and BCG	61.88 ± 10.63 ^e^	439.8333	15 (5–63) ^d^	Multivariate	RFS	7	(18)
18	Liu, et al.	2022	China	Retrospective	183	BC	TURBT and BCG	62.37 ± 13.34 ^e^	514.4732	30 (13.0–47.0) ^b^	Multivariate	RFS	7	(19)
19	Zhang, et al.	2022	China	Retrospective	725	BC	RC	65 (59-72) ^b^	554.23	36(OS) ^a^; 33.6(RFS) ^a^	Multivariate	OS、RFS	8	(30)
20	Li, et al.	2022	China	Retrospective	197	BC	BCG	64.17 ± 11.05 ^e^	557	30.18 ± 15.67 ^e^	Multivariate	RFS、PFS	7	(31)
21	Ding, et al.	2022	China	Retrospective	416	BC	TURBT	67 (58, 75) ^b^	505	21 (14.75– 32) ^b^	Multivariate	RFS	8	(32)

UC, Urothelial carcinoma; UTUC, Upper tract urothelial carcinoma; BC, Bladder cancer; RNU, Radical nephroureterectomy; TURBT, Transurethral resection of bladder tumor; RC, Radical cystectomy; BCG, Bacillus Calmette -Guerin; M, Median; I, Interquartile range; R, Range; NR, Not reported; OS, Overall Survival; CSS, Cancer-specific survival; RFS, Recurrence-free survival; PFS, Progression-free survival.

a Median.

b Median (interquartile range).

c Mean(rang).

d Median (rang).

e Mean.

### Prognostic significance of SII on OS in patients with UC

**Figure 2:**
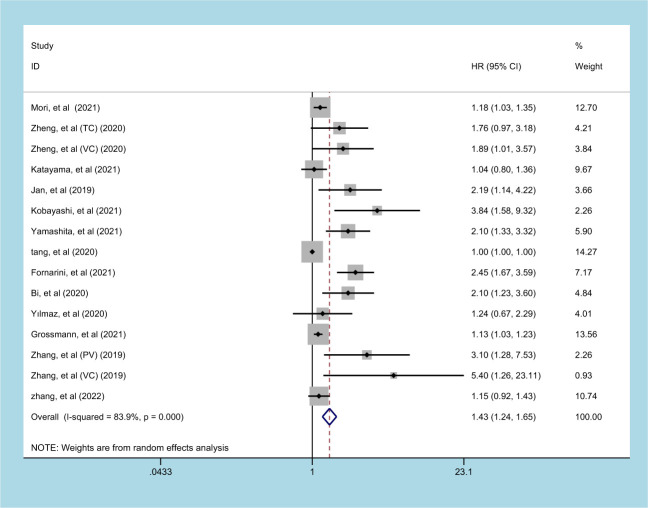
Forest plot and meta-analysis of the relationship between OS and SII.

13 studies (involving 15 datasets) involving 10933 patients reported an association between preoperative SII and OS in patients with UC (11, 13–15, 20, 22–25, 27–30). The pooled analysis indicated that patients with an increased preoperative SII had a significantly worse OS (HR=1.430, 95% CI 1.237-1.653, *p*<0.001), with significant heterogeneity between studies (I^2^ = 83.9%, *p*<0.001) (**Figure 2**, **Table 2**). High SII was also significantly associated with poor OS in the subgroup of ethnicity, carcinoma type, cutoff value, and sample size (*p*<0.05) (**Table 2**).

**Table 2:** Subgroup analyses of OS, CSS, RFS, and PFS.

Outcome	Variable	No. of datasets	HR (95% CI)	*p*	Heterogeneity		References
					I^2^ (%)	*P*	
**OS**	**All**	**15**	**1.430(1.237-1.653)**	**<0.001**	**83.9**	**<0.001**	(11, 13–15, 20, 22–25, 27–30)
Ethnicity	Asian	10	1.830(1.355-2.471)	<0.001	82.7	<0.001	(13–15, 20, 23, 27, 28, 30)
	Caucasian	5	1.261(1.046-1.520)	0.015	74.6	0.003	(11, 22, 24, 25, 29)
Tumor type	UTUC	5	1.810(1.200-2.731)	<0.001	67.9	0.014	(11, 15, 23, 27)
	Mixed	1	2.450(1.671-3.592)	<0.001	–	–	(22)
	BC	9	1.233(1.064-1.429)	0.005	79.1	<0.001	(13, 14, 20, 24, 25, 28–30)
Sample size	≤250	6	2.008(1.168-3.453)	0.012	83.8	<0.001	(13–15, 25, 28)
	>250	9	1.383(1.171-1.632)	<0.001	70.9	0.001	(11, 20, 22–24, 27, 29, 30)
Cutoff value	≤520	6	2.228(1.390-3.570)	0.001	77.5	<0.001	(11, 14, 15, 20, 28)
	>520	9	1.264(1.089-1.468)	0.002	81.6	<0.001	(13, 22–25, 27, 29, 30)
**CSS**	**All**	**10**	**1.913(1.473-2.485)**	**<0.001**	**73.5**	**<0.001**	(11, 15, 20, 21, 23, 24, 27–29)
Ethnicity	Asian	7	2.395(1.795-3.195)	<0.001	11.5	0.342	(15, 20, 21, 23, 27, 28)
	Caucasian	3	1.301(1.046-1.619)	0.018	69.2	0.039	(11, 24, 29)
Tumor type	UTUC	6	2.612(1.451-4.700)	0.001	77.0	0.001	(11, 15, 21, 23, 27)
	Mixed	–	–	–	–	–	–
	BC	4	1.676(1.139-2.465)	0.009	73.5	0.011	(20, 24, 28, 29)
Sample size	≤250	2	3.278(1.006-10.683)	0.049	73.4	0.052	(15, 28)
	>250	8	1.724(1.337-2.223)	<0.001	70.0	0.002	(11, 20, 21, 23, 24, 27, 29)
Cutoff value	≤520	5	2.033(1.273-3.248)	0.003	74.7	0.003	(11, 15, 20, 21, 28)
	>520	5	2.126(1.258-3.593)	0.005	77.2	0.002	(23, 24, 27, 29)
**RFS**	**All**	**12**	**1.240(1.097-1.403)**	**0.001**	**78.0**	**<0.001**	(11, 13, 18, 19, 23, 24, 26, 27, 29, 30, 32)
Ethnicity	Asian	9	1.320(1.063-1.641)	0.012	78.2	<0.001	(13, 18, 19, 23, 26, 27, 30, 32)
	Caucasian	3	1.159(1.070-1.256)	<0.001	0.0	0.741	(11, 24, 29)
Tumor type	UTUC	4	1.245(1.087-1.427)	0.002	0.0	0.499	(11, 23, 27)
	Mixed	–	–	–	–	–	–
	BC	8	1.209(1.045-1.399)	0.011	81.5	<0.001	(13, 18, 19, 24, 26, 29, 30, 32)
Sample size	≤250	4	1.154(0.788-1.689)	0.463	82.7	0.304	(13, 18, 19, 26)
	>250	8	1.221(1.119-1.332)	<0.001	16.0	0.001	(11, 23, 24, 27, 29, 30, 32)
Cutoff value	≤520	5	1.332(0.980-1.811)	0.067	74.2	0.004	(11, 18, 19, 26, 32)
	>520	7	1.158(1.029-1.304)	0.015	68.4	0.004	(13, 23, 24, 27, 29, 30)
**PFS**	**All**	**4**	**1.844(1.488-2.284)**	**<0.001**	**0.0**	**0.929**	(22, 24, 25, 31)
Ethnicity	Asian	1	1.978(1.091-3.588)	0.025	–	–	(31)
	Caucasian	3	1.825(1.450-2.296)	<0.001	0.0	0.822	(22, 24, 25)
Tumor type	UTUC	–	–	–	–	–	–
	Mixed	1	1.910(1.389-2.627)	<0.001	–	–	(22)
	BC	3	1.791(1.342-2.392)	<0.001	0.0	0.832	(24, 25, 31)
Sample size	≤250	2	1.742(1.154-2.630)	0.008	0.0	0.563	(22, 24)
	>250	2	1.883(1.465-2.420)	<0.001	0.0	0.887	(25, 31)
Cutoff value	≤520	–	–	–	–	–	–
	>520	4	1.844(1.488-2.284)	<0.001	0.0	0.929	(22, 24, 25, 31)

### Prognostic significance of SII on CSS in patients with UC

**Figure 3:**
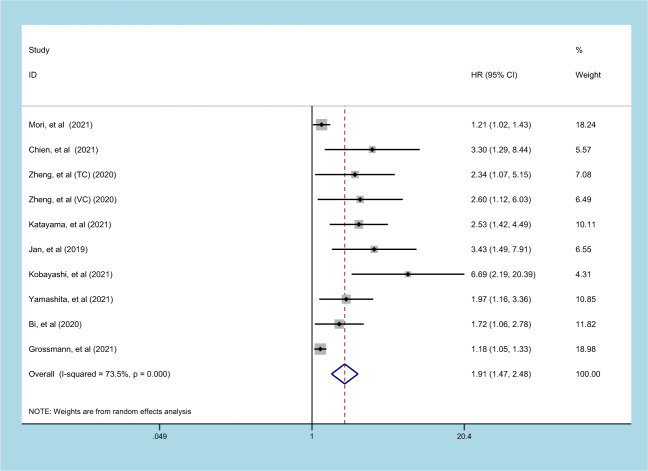
Forest plot and meta-analysis of the relationship between CSS and SII.

9 studies (involving 10 datasets) comprising 9877 patients reported on the prognostic effect of preoperative SII on CSS in UC patients (11, 15, 20, 21, 23, 24, 27–29). The pooled analysis demonstrated that higher preoperative SII in UC patients was an independent predictor of CSS (HR=1.913, 95% CI 1.473-2.485, *p*<0.001), with significant heterogeneity (I^2^ = 73.5%, *p*<0.001) (**Figure 3**, **Table 2**). Furthermore, an elevated SII was significantly associated with inferior CSS in patients with subgroups of ethnicity, carcinoma type, cutoff value, and sample size (*p*<0.05) (**Table 2**).

### Prognostic significance of SII on RFS in patients with UC

**Figure 4:**
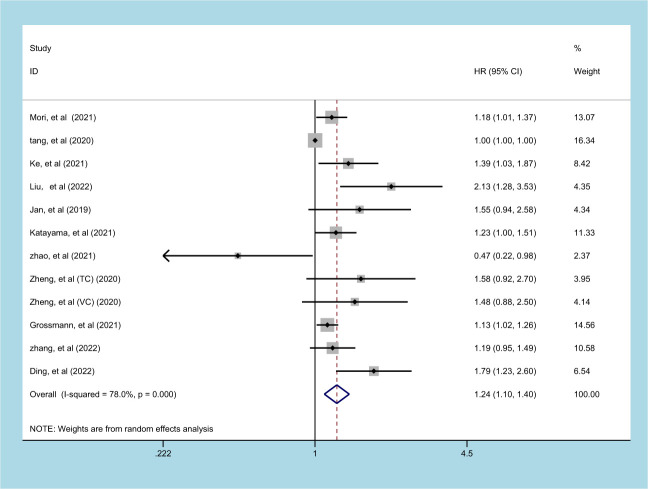
Forest plot and meta-analysis of the relationship between RFS and SII.

11 studies (involving 12 datasets) comprising 10577 patients reported on the prognostic effect of preoperative SII on RFS in UC patients (11, 13, 18, 19, 23, 24, 26, 27, 29, 30, 32). The pooled analysis demonstrated that higher preoperative SII in UC patients was an independent predictor of RFS (HR = 1.240, 95% CI 1.097-1.403, p=0.001), with significant heterogeneity (I^2^ = 78.0%, p<0.001) (**Figure 4**, **Table 2**). An elevated SII was significantly associated with inferior RFS in patients with ethnicity and carcinoma type (*p*<0.05). However, there was no statistical significance in the sample size of less than 250 (*p*=0.463) and cutoff value ≤ 520 (*p*=0.067) (**Table 2**).

### Prognostic significance of SII on PFS in patients with UC

**Figure 5:**
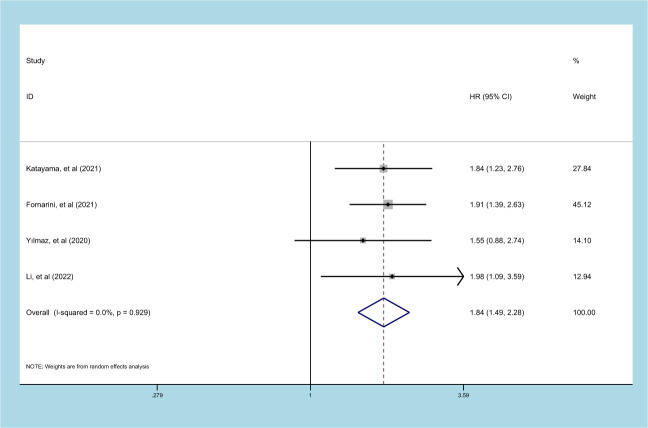
Forest plot and meta-analysis of the relationship between PFS and SII.

4 studies comprising 1733 patients reported on the prognostic effect of preoperative SII on PFS in UC patients (22, 24, 25, 31). The pooled analysis demonstrated that higher preoperative SII in UC patients was an independent predictor of PFS (HR=1.844, 95% CI 1.488-2.284, *p*<0.001), without heterogeneity (I^2^ = 0, *p*=0.929) (**Figure 5**, **Table 2**). An elevated SII was significantly associated with inferior PFS in patients with ethnicity, carcinoma type, cutoff value and sample size (*p*<0.05) (**Table 2**).

### Sensitivity analysis

Sensitivity analyses were conducted to evaluate the reliability of pooled HRs for OS, CSS, RFS and PFS, and to avoid the influence of low-quality studies on the results of the meta-analyses. The leave-one-out test showed no significant change in the overall HR estimates for these survival outcomes, indicating the results of these meta-analyses were stable, as shown in **Figure S1**.

### Publication bias

Begg’s test and funnel plot were used to assess the publication bias in included studies. The results of these tests were not statistically significant (OS: *p* = 0.488, RFS: *p* = 0.283, PFS: *p* = 1.000), except CSS: *p*= 0.007. Visual examination of funnel plot showed asymmetry, which increased the possibility of potential publication bias (**Figure S2**).

## Discussion

BC accounted for 5% of the total carcinoma costs in the European Union, with higher-income countries spending higher budgets on this carcinoma (33). UTUC accounts for just 5–10% of all urothelial malignancies with a poor prognosis (1). Microscopic or gross hematuria can be an early sign of UC. However, it was often terminal stage when the patient was diagnosed. Another problem in UC is the high recurrence rate, which means that patients need lifelong postoperative monitoring, which is unbearable to most UC patients (34). Therefore, biomarkers are needed to detect relapse and develop treatment plans. The ideal biomarkers are inexpensive and readily available, which can better help clinicians develop appropriate individual treatment plans and postoperative follow-up plans for different UC patients. To our knowledge, inflammation and immune responses are thought to be factors in the development and progression of malignant carcinoma. The two influence each other to maintain a dynamic balance, once the balance is broken, it will cause the occurrence of carcinoma, thus promoting the proliferation and invasion of carcinoma cells. This process requires the participation of platelets, neutrophils, and lymphocytes, so the evaluation value of SII on immune inflammation is considered feasible, and its predictive value in carcinoma has been fully verified in lung carcinoma, bile duct carcinoma, gastric carcinoma, et al (35, 36). Therefore SII stands out from many biomarkers as an easy and inexpensive indicator. Katayama and his colleagues first reported that SII could predict disease progression and survival of UC in 2018 (23). It was immediately confirmed by Zhang and Zheng (14, 27). However, studies in Turkey and China tell a different story (13, 25), considering it meaningless or meaningful in a given situation. This inconsistency in poor prognostic consistency brings confusion to clinical application.

This is the first evidence-based study that fully analyzed the prognostic value of SII for predictive value for OS, CSS, PFS and RFS of UC patients. A total of 19 published studies explored the prognostic and survival indicators of SII in UC patients. Our meta-analysis demonstrated that higher SII levels were associated with worse OS, CSS, PFS, and RFS in UC patients, indicating that SII is a crucial prognostic indicator for UC. These findings align with previous studies (14, 27) and provide a comprehensive evaluation of the potential of SII as a predictor for UC prognosis. Sensitivity analysis indicates that the results have good stability and reliability. When conducting subgroup analysis based on different influencing factors, the results for OS, CSS, RFS, and PFS were found to be very similar. However, it is important to note that the subgroup analysis cannot identify the source of heterogeneity, and therefore, the results should be interpreted with caution.

Previously, three meta-analyses were reported on the predictive value of SII for prognosis in urinary system carcinoma, which did not specifically address the condition of urothelium. One study included all urinary carcinoma, included renal cell carcinoma and testicular carcinoma (37). The other two studies only focus on bladder carcinoma (38, 39), ignoring the similarities in morphological structure and carcinoma biology of urinary transitional epithelium (40). This similarity not only supports the conclusions of this meta-analysis but also provides some enlightenment from it. Carcinoma type is not a factor that limits the realization of SII. This conclusion fully assess the discriminatory power of the SII as a biomarker in UC. Besides, previous studies have focused on the predictive value of 2-3 indicators, our study contributes to the growing body of literature on prognostic indicators by highlighting the importance of considering 4 factors when predicting clinical outcomes. Specifically, our findings demonstrate a significant correlation between four prognostic indicators and the clinical outcomes of the subjects. These results suggest that a multi-indicator approach may provide more accurate prognostic guidance and improve clinical decision-making.

SII changes continuously with the course of treatment, and the prognosis of the disease can be predicted before and after treatment (31). To better understand this course, it is necessary to collect SII data systematically on a regular and larger scale. Identifying the prognostic value of preoperative SII, SII during induction and their dynamic change, and comparing the prognostic value of these 3 factors are needed in the future. When necessary, the courses were analyzed jointly with Neutrophil to lymphocyte ratio (13), abdominal fat distribution (18), sarcopenia (19) and other indicators. A high SII was found to be an independent prognostic factor for worse RFS in UC patients with high blood pressure, diabetes mellitus and without peripheral nerve invasion (30). Therefore, it is necessary to identify which subgroups of UC patients may benefit more from SII assessment and more accurately predict survival outcomes.

A funnel plot is an intuitive method for assessing publication bias, but not all instances of funnel plot asymmetry are solely due to publication bias. When there is substantial heterogeneity among studies (I^2^ > 75%), funnel plots may exhibit significant asymmetry, among other reasons. In contrast to the intuitive impression of funnel plots, Begg’s test is a more precise tool for detecting the presence of publication bias. However, Begg’s test has lower sensitivity when the number of studies is limited. Both methods have their own limitations to varying degrees, so we employed both testing approaches simultaneously. Given the relatively small number of studies included in this paper and the substantial heterogeneity among them, we still suspect the presence of potential publication bias.

However, this study also has some limitations. The first limitation is all the included studies were retrospective, with a low level of evidence. Next the cut-off values of SII given in the literature included in this study are different, and the standards and methods of cut-off values of different research results are different. We could not come up with a unique cut-off through statistical analysis. This can affect results and lead to inevitable potential heterogeneity and bias. Despite limitations, our results give us some hints that can help facilitate clinicians in administering further adjuvant therapies and having closer follow-up.

## Conclusions

Preoperative SII can serve as a reliable biomarker for predicting the survival and disease recurrence of UC patients. The higher SII is in UC patients, the worse OS, CSS, RFS and PFS are. However, the conclusions are based on evidence-based research with high heterogeneity, and further prospective studies are needed to confirm the clinical utility of SII in UC management.

## Data availability statement

The original contributions presented in the study are included in the article/**Supplementary Material**. Further inquiries can be directed to the corresponding authors.

## Author contributions

JZ: Methodology, Writing – original draft. LP: Data curation, Methodology, Writing – original draft. SZ: Funding acquisition, Writing – review & editing. HL: Data curation, Software, Writing – review & editing. JH: Data curation, Formal Analysis, Software, Writing – review & editing. HS: Funding acquisition, Conceptualization, Writing – review & editing. SW: Conceptualization, Funding acquisition, Writing – review & editing.

## Supplementary material

Supplementary Figure 1 Sensitivity analysis. **(A)** Sensitivity analysis forest plot for OS; **(B)** Sensitivity analysis forest plot for CSS; **(C)** Sensitivity analysis forest plot for RFS; **(D)** Sensitivity analysis forest plot for PFS.

Supplementary Figure 2 Publication bias analysis. **(A)** Publication bias funnel plot for OS; **(B)** Publication bias funnel plot for CSS; **(C)** Publication bias funnel plot for RFS; **(D)** Publication bias funnel plot for PFS.
