# Supplementary figures and images for: Preoperative systemic immune-inflammation index as a prognostic indicator for patients with urothelial carcinoma
Source: Front Immunol. 2023 Nov 20;14:1275033. doi: 10.3389/fimmu.2023.1275033 (PMC10694229; doi:10.3389/fimmu.2023.1275033)

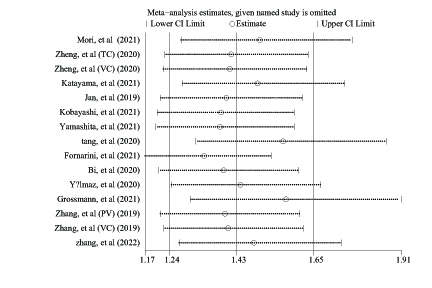

Supplement: Supplementary Figure 1 — Sensitivity analysis. (A) Sensitivity analysis forest plot for OS; (B) Sensitivity analysis forest plot for CSS; (C) Sensitivity analysis forest plot for RFS; (D) Sensitivity analysis forest plot for PFS. [file DataSheet_1.zip › Figure S1/Figure S1-1.JPEG]

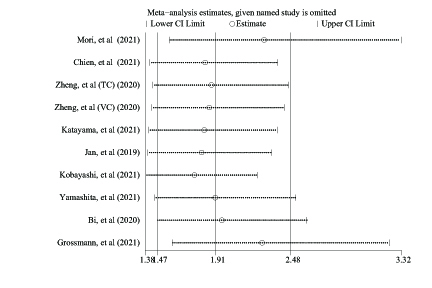

Supplement: Supplementary Figure 1 — Sensitivity analysis. (A) Sensitivity analysis forest plot for OS; (B) Sensitivity analysis forest plot for CSS; (C) Sensitivity analysis forest plot for RFS; (D) Sensitivity analysis forest plot for PFS. [file DataSheet_1.zip › Figure S1/Figure S1-2.JPEG]

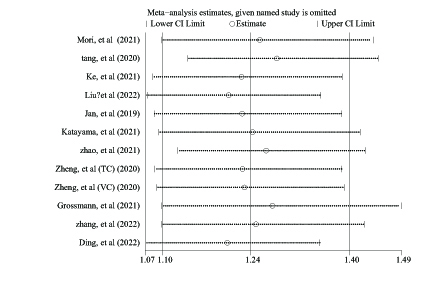

Supplement: Supplementary Figure 1 — Sensitivity analysis. (A) Sensitivity analysis forest plot for OS; (B) Sensitivity analysis forest plot for CSS; (C) Sensitivity analysis forest plot for RFS; (D) Sensitivity analysis forest plot for PFS. [file DataSheet_1.zip › Figure S1/Figure S1-3.JPEG]

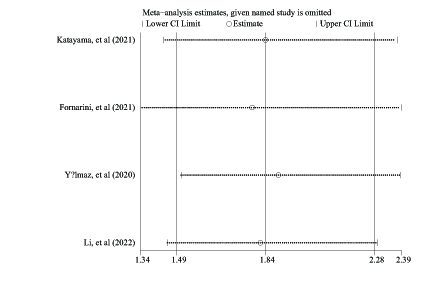

Supplement: Supplementary Figure 1 — Sensitivity analysis. (A) Sensitivity analysis forest plot for OS; (B) Sensitivity analysis forest plot for CSS; (C) Sensitivity analysis forest plot for RFS; (D) Sensitivity analysis forest plot for PFS. [file DataSheet_1.zip › Figure S1/Figure S1-4.JPEG]

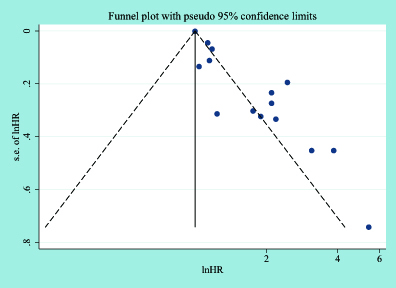

Supplement: Supplementary Figure 2 — Publication bias analysis. (A) Publication bias funnel plot for OS; (B) Publication bias funnel plot for CSS; (C) Publication bias funnel plot for RFS; (D) Publication bias funnel plot for PFS. [file DataSheet_2.zip › Figure S2/Figure S2-1.JPEG]

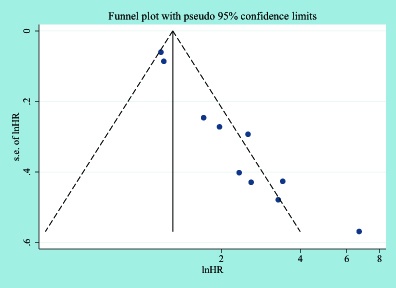

Supplement: Supplementary Figure 2 — Publication bias analysis. (A) Publication bias funnel plot for OS; (B) Publication bias funnel plot for CSS; (C) Publication bias funnel plot for RFS; (D) Publication bias funnel plot for PFS. [file DataSheet_2.zip › Figure S2/Figure S2-2.JPEG]

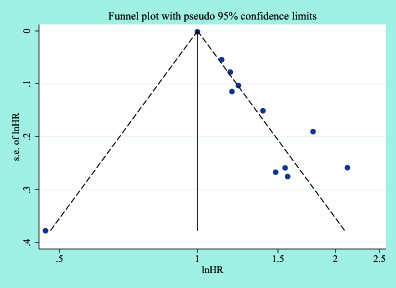

Supplement: Supplementary Figure 2 — Publication bias analysis. (A) Publication bias funnel plot for OS; (B) Publication bias funnel plot for CSS; (C) Publication bias funnel plot for RFS; (D) Publication bias funnel plot for PFS. [file DataSheet_2.zip › Figure S2/Figure S2-3.JPEG]

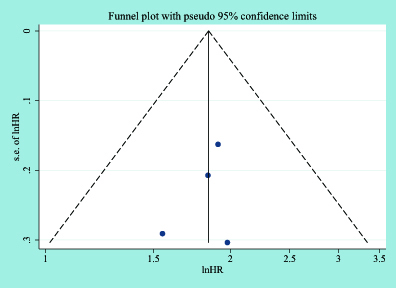

Supplement: Supplementary Figure 2 — Publication bias analysis. (A) Publication bias funnel plot for OS; (B) Publication bias funnel plot for CSS; (C) Publication bias funnel plot for RFS; (D) Publication bias funnel plot for PFS. [file DataSheet_2.zip › Figure S2/Figure S2-4.JPEG]
